# Neuroventilatory efficiency and extubation readiness in critically ill patients

**DOI:** 10.1186/cc11451

**Published:** 2012-07-31

**Authors:** Ling Liu, Huogen Liu, Yi Yang, Yingzi Huang, Songqiao Liu, Jennifer Beck, Arthur S Slutsky, Christer Sinderby, Haibo Qiu

**Affiliations:** 1 Department of Critical Care Medicine, Nanjing Zhong-Da Hospital, Southeast University School of Medicine, 87 Dingjiaqiao Street, Nanjing 210009, China; 2 Department of Critical Care, St. Michael's Hospital, Keenan Research Centre in the Li Ka Shing Knowledge Institute of St. Michael's Hospital, 30 Bond Street, Toronto, Ontario, M5B1W8 Canada; 3 Department of Pediatrics, University of Toronto, Hospital for Sick Children, 555 University Avenue, Toronto, Ontario M5G 1X8 Canada; 4 Department of Medicine, Interdepartmental Division of Critical Care Medicine, University of Toronto, Ontario M5G 1X8 Canada

## Abstract

**Introduction:**

Based on the hypothesis that failure of weaning from mechanical ventilation is caused by respiratory demand exceeding the capacity of the respiratory muscles, we evaluated whether extubation failure could be characterized by increased respiratory drive and impaired efficiency to generate inspiratory pressure and ventilation.

**Methods:**

Airway pressure, flow, volume, breathing frequency, and diaphragm electrical activity were measured in a heterogeneous group of patients deemed ready for a spontaneous breathing trial. Efficiency to convert neuromuscular activity into inspiratory pressure was calculated as the ratio of negative airway pressure and diaphragm electrical activity during an inspiratory occlusion. Efficiency to convert neuromuscular activity into volume was calculated as the ratio of the tidal volume to diaphragm electrical activity. All variables were obtained during a 30-minute spontaneous breathing trial on continuous positive airway pressure (CPAP) of 5 cm H_2_O and compared between patients for whom extubation succeeded with those for whom either the spontaneous breathing trial failed or for those who passed, but then the extubation failed.

**Results:**

Of 52 patients enrolled in the study, 35 (67.3%) were successfully extubated, and 17 (32.7%) were not. Patients for whom it failed had higher diaphragm electrical activity (48%; *P *< 0.001) and a lower efficiency to convert neuromuscular activity into inspiratory pressure and tidal volume (40% (*P *< 0.001) and 53% (*P *< 0.001)), respectively. Neuroventilatory efficiency demonstrated the greatest predictability for weaning success.

**Conclusions:**

This study shows that a mixed group of critically ill patients for whom weaning fails have increased neural respiratory drive and impaired ability to convert neuromuscular activity into tidal ventilation, in part because of diaphragm weakness.

**Trial Registration:**

Clinicaltrials.gov identifier NCT01065428. ©2012 Liu et al.; licensee BioMed Central Ltd. This is an open access article distributed under the terms of the Creative Commons Attribution License (http://creativecommons.org/licenses/by/2.0), which permits unrestricted use, distribution, and reproduction in any medium, provided the original work is properly cited.

## Introduction

Mechanical ventilation is essential for patients with acute respiratory failure, yet it is associated with risks and complications that prolong its duration and increase the risk of death [1]. Mechanically ventilated patients can develop rapid and severe diaphragm weakness [2-8]. Compared with successfully weaned patients, patients for whom weaning fails have increased respiratory loads [9] and demonstrate increased respiratory pressures during spontaneous breathing trials (SBTs) [9-11]. This scenario agrees well with the hypothesis that weaning failure is partly due to the patients' respiratory loads exceeding the capacity of their respiratory muscles [12-16]. If this is correct, then difficult-to-wean patients would be expected to demonstrate increased respiratory drive and compromised ventilatory breathing efficiency.

The introduction of Neurally Adjusted Ventilatory Assist (NAVA) [17] has made available a standardized and validated method to monitor and measure diaphragm electrical activity (EA_di_) [18] during conventional modes of ventilation as well as during spontaneous breathing trials. The EA_di _allows quantification of the neural respiratory drive to the diaphragm [19,20]. If expressed in relation to airway pressure (P_aw_) during an inspiratory effort against an occluded respiratory circuit, the P_aw_/EA_di _ratio provides an estimate of inspiratory pressure generation normalized to neural inspiratory effort (that is, the neuromechanical efficiency (NME)) [21]. If the tidal volume (V_t_) is expressed in relation to EA_di_, the V_t_/EA_di _ratio expresses the ability to generate volume normalized to neural drive (that is, the neuroventilatory efficiency (NVE)). A recent study suggests that these physiological indices (EA_di_, NME, and NVE) may predict readiness for extubation in chronic obstructive pulmonary disease (COPD) patients [22].

The aim of this study was to examine whether a general patient population for whom extubation fails, when compared with successfully extubated patients, have increased EA_di_, reflecting a higher neural respiratory drive, have lowered NME as an indication of more pronounced respiratory muscle weakness, and have a reduction in NVE due to a combination of weakness and increased load.

## Materials and methods

The trial was conducted in a 20-bed general intensive care unit (ICU) of a teaching hospital affiliated with Southeast University in China. The protocol was approved by Jiangsu Institutional Ethics Committee (Approval Number: 2009ZDLL012.0), and written informed consent was obtained from the patients or next of kin. The trial was registered at clinicaltrials.gov (NCT01065428).

**Figure 1:**
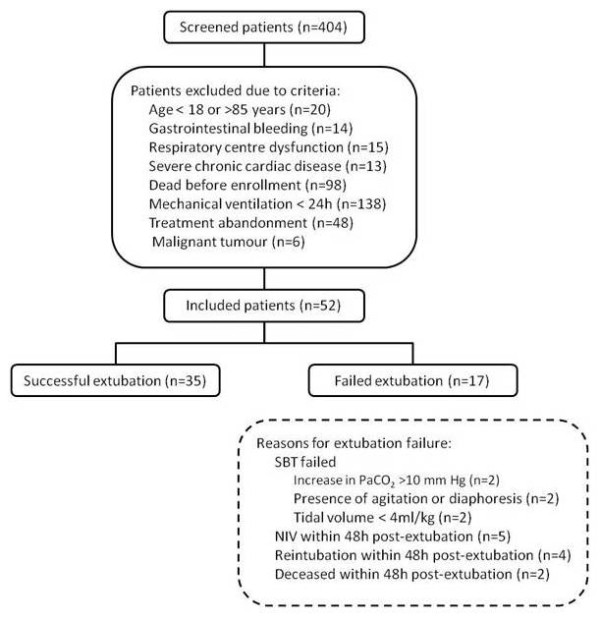
**Flow chart of the study**.

Intubated patients receiving controlled mechanical ventilation for >24 hours were screened and were enrolled if they did not meet exclusion criteria (Figure 1). The enrolled patients were switched to a Servo-i ventilator (Maquet, Solna, Sweden), and the conventional nasogastric tube was replaced by one capable of measuring the EA_di _(Maquet). Patients were then ventilated with pressure support (PSV) or synchronized intermittent mandatory ventilation (SIMV) + PSV. The following morning, patients were screened by the clinical team, and an SBT was performed if (a) the cause of mechanical ventilation was resolved, (b) PaO_2_/F_i_O_2 _> 200; PEEP ≤5 cm H_2_O; F_i_O_2 _≤50%; and respiratory frequency (f) <35 breath/min; (c) patients were hemodynamically stable (heart rate <140 beats/min, no vasopressors required, or <5 μg/kg/min dopamine); (d) no sedation or receiving minimal sedation with a low dose of morphine (<3 mg/h, by continuous intravenous infusion); and (e) patients were breathing spontaneously with adequate cough. Cough was evaluated by placing a white card about 1.5 cm away from the end of the endotracheal tube and asking the patient to cough (3 to 4 times). Adequate cough was considered if wetness appeared on the card [23]. If the criteria were not fulfilled, a new screening was performed the next morning.

The protocol started with patients ventilated on a PSV of 10 cm H_2_O above a positive end-expiratory pressure (PEEP) of 5 cm H_2_O (PSV_10_) for 5 minutes. Then assist was removed, and a 30-minute SBT was performed with continuous positive airway pressure (CPAP) of 5 cm H_2_O at the prescribed level of F_i_O_2_. At 1, 5, 10, 15, and 30 minutes into the SBT, inspiratory occlusions (starting at end expiration) were performed for one or two breath cycles by using the internal Servo-i feature.

Patients who reached the criteria for failed SBT: (a) impaired gas exchange (SpO_2 _<90%; PaO_2 _<60 mm Hg; increase in PaCO_2 _>10 mm Hg); (b) hemodynamic instability (heart rate changed >20%; systolic blood pressure (BP) >180 or <90 mm Hg; BP changed >20%; vasopressors required); (c) unstable ventilatory pattern (>35 breaths/min; V_t _<4 ml/kg); (d) change in mental status (somnolence, coma, agitation, anxiety), diaphoresis, and other onset or worsening of discomfort deemed by the clinical team, resumed mechanical ventilation. Patients who were able to tolerate the 30-minute SBT without reaching the exclusion criteria were extubated. Patients who were extubated from a completed SBT and remained extubated for more than 48 hours were considered to be successfully extubated [24].

Patients who resumed mechanical ventilation during the SBT, or required noninvasive ventilation (NIV) after extubation, or were reintubated, or deceased within 48 hours after extubation were considered to have failed extubation. The criteria for reintubation were (a) emergency status, such as respiratory or cardiac arrest, and gasping for air; (b) neurologic deterioration (coma or agitation requiring continuous intravenous sedation); (c) hemodynamic instability (that is, need for continuous infusion of epinephrine, norepinephrine, or dopamine (>5 µg/kg/min) to maintain systolic arterial pressure >90 mm Hg); (d) upper airway obstruction; (e) unmanageable tracheobronchial secretions; and (f) respiratory distress, as assessed by the combination of SpO_2 _<90% (SpO_2 _<85% for COPD patients), respiratory rate >35/min, and visible accessory muscle recruitment or thoracoabdominal paradox, despite administration of oxygen and noninvasive ventilation.

In case of extubation failure, NIV was administered through an oronasal mask when an F_i_O_2 _of 0.4 failed to maintain SpO_2 _≥90% (SpO_2 _≥85% for COPD patients), and the patient showed evidence of respiratory distress, as assessed by the combination of tachypnea (>30 breaths/min) and visible accessory muscle recruitment.

Throughout the protocol, EA_di_, P_aw_, flow, V_T_, f, and minute ventilation (V_E_) were acquired from the Servo-I ventilator into a personal computer, by using "Servo-tracker" software (Maquet). Arterial blood gases were measured at the end of PSV_10 _and at 5 and 30 minutes (or at termination) of the SBT.

NME was calculated as the ratio of the (P_aw_-PEEP) divided by EA_di _during inspiratory occlusion. NVE was calculated as the ratio of V_t _and EA_di _during inspiration. All parameters and indices (except NME) were calculated as the mean value of five inspirations at each time point. NVE was calculated from one inspiratory occlusion at each time point.

### Statistics

Statistical analysis was performed with SPSS 13.0 (IBM, Minneapolis, MN, USA) and SigmaStat 3.5 (Systat Software Inc., Chicago, IL, USA). Results are presented as mean ± SD, and significant difference was defined as *P *< 0.05. A *t *test for independent variables and χ^2 ^tests were used to compare group characteristics and causes of ARF. Two-way repeated measures ANOVA with Student-Newman-Keuls *post hoc *comparison test was used to compare variables between groups and time points. Spearman correlation and linear regression analysis was used to determine relations between variables. ROC (receiver operating characteristic) curves were generated, and the area under the curve (AUC) was calculated, as well as sensitivity, specificity, and the Youden index, to determine the utility of measured variables to predict extubation outcome. Generalized *U *statistics were used to compare areas under the ROC curves [25].

## Results

In total, 404 patients were screened; 352 patients met exclusion criteria, and 52 patients were eligible (Figure 1). Of the 52 patients enrolled, 35 (67.3%) were successfully extubated, and for 17 (32.7%), extubation failed. Two extubation-failure patients died because of refusal to reintubate by the family.

All patients discontinued sedative drugs (propofol, midazolam) at least 1 hour before the SBT. Morphine delivery was discontinued in nine (25.7%) and six (35.3%) of the successfully extubated and failed-extubation groups (NS), respectively. In the remainder of the patients, low-dose morphine infusion was 1.1 ± 0.5 mg/h and 1.2 ± 0.5 mg/h for the successfully extubated and failed-extubation groups (NS), respectively. Clinical characteristics of the patients are presented in Table 1.

**Table 1:** Patient description

	Successful extubation (*n *= 35)	Failed extubation (*n *= 17)	*P *value
Age, years (mean ± SD)	65.2 ± 18.5	76.0 ± 6.4	0.023
Sex, male/female, *n*	24/11	8/9	0.263
Ideal body weight, kg (mean ± SD)	56.7 ± 9.1	58.4 ± 8.4	0.501
Days on ventilator, days (mean ± SD)	3.4 ± 2.7	4.5 ± 3.7	0.284
Causes of ARF			
COPD exacerbation, *n *(%)	8 (22.9)	10 (58.8)	0.011
Pneumonia, *n *(%)	9 (25.7)	6 (35.3)	0.697
Sepsis with ALI, *n *(%)	3 (8.6)	0 (0)	0.542
Multitrauma, *n *(%)	3 (8.6)	0 (0)	0.542
Postoperative, *n *(%)	6 (17.1)	0 (0)	0.176
Other, *n *(%)	6 (17.1)	1 (5.9)	0.495

ALI, acute lung injury; ARF, acute respiratory failure; COPD, chronic obstructive pulmonary disease; SD, standard deviation.

### EA_di_, NVE, and NME

**Figure 2:**
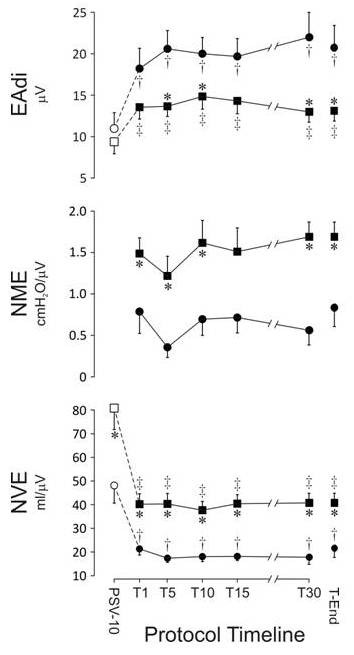
**The development of (top to bottom) diaphragm electrical activity (EA_di_), neuromechanical efficiency (NVE), and neuroventilatory efficiency (NME) during pressure support of 10 above 5 cm H_2_O of PEEP (PSV_10_) and throughout the spontaneous breathing trial (SBT) on CPAP of 5 cm H_2_O**. On the X-axis: T_1_, T_5_, T_10_, T_15_, T_30_, and T_end _indicate minutes 1, 5, 10, 15, and 30, as well as the last data obtained during the SBT. In the successfully extubated group, *n *= 35 at all time points. In the group for whom extubation failed, n = 17 at PSV_10_, T_1_, and T_5_; *n*=16 at T_10 _and T_15_; *n*=14 at T_30 _(three for whom SBT failed at 30 minutes and 11 who were initially extubated but were later reintubated or provided with noninvasive ventilation or died). Data were obtained for the remaining three patients meeting SBT exclusion criteria at T_30_. At T_end_, *n *= 17 in the group for whom extubation failed. *Difference (*P *< 0.05) between failure and successfully extubated groups at the same time point. †Differences (*P *< 0.05) between PSV_10 _and other time points in the group for whom extubation failed. ‡Differences (*P *< 0.05) between PSV_10 _and other time points in the group that was successfully extubated.

The progression of EA_di_, NVE, and NME from PSV_10 _and throughout the SBT are depicted in Figure 2 for both the successful and the failed-extubation groups.

During PSV_10_, EA_di _was approximately 10 μV and was not different between the success and failure groups. During the SBT, the EA_di _increased significantly in both groups and reached a plateau during the first few minutes. The increase in EA_di _in the extubation-failure group was twice that observed in the successfully extubated group.

The NVE values at PSV_10 _were about 80 and 50 ml/μV in the success and failure groups, respectively. At the onset of the SBT, the NVE decreased significantly in both groups; thereafter, the NVE for both groups reached a plateau at about 40 and 20 ml/μV in the success and failure patients, respectively.

NVE was inversely correlated with age, both for all subjects (*r *= 0.58 ± 0.04; *P *< 0.001; *n *= 52) and for the successfully extubated group (*r *= 0.53 ± 0.05; *P *= 0.007; *n *= 35). No relation was found between EA_di_, NME, or NVE and duration of mechanical ventilation.

NME was not obtained during PSV_10_. During the SBT, NME was significantly higher in the success group (~1.5 cm H_2_O/μV) compared with the failure group (~1.0 cm H_2_O/μV); NME did not change significantly with time.

Subanalysis of the patients for whom extubation failed revealed no significant difference for EA_di_, NVE, and NME between those for whom the SBT failed (*n *= 6) and those who were extubated but required reintubation, NIV, or died within 48 hours (*n *= 11). Nor was a difference noted at any time point for EA_di_, NVE, and NME between COPD and non-COPD patients in either the success or failure groups.

Regression analysis showed that 40% (*r *= 0.67; *P *< 0.001) and 45% (*r *= 0.64; *P *< 0.001) of the changes in V_t _and V_t_/kg IBW (ideal body weight) from T_1 _to T_end _were explained by simultaneous changes of the EA_di _from T_1 _to T_end_. EA_di _was not correlated with f or f/V_t_.

### Breathing pattern

**Figure 3:**
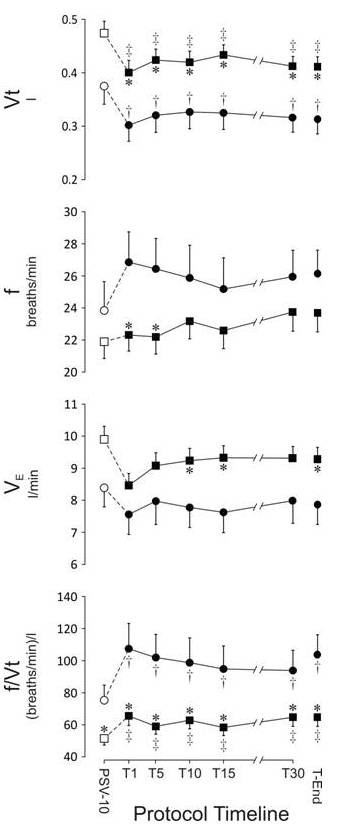
**The development of (top to bottom) of tidal volume (V_t_), breathing frequency (f), minute ventilation (V_E_), and the ratio of breathing frequency and tidal volume (f/V_t_) during pressure support of 10 above 5 cm H_2_O of PEEP (PSV_10_) and throughout the spontaneous breathing trial (SBT) on CPAP of 5 cm H_2_O**. See Figure 2 for more description.

Figure 3 shows the timelines of V_t_, f, V_E_, and f/V_t_.

During the PSV_10 _period, V_t _was 474 ml (8.1 ml/kg IBW) in the group that was successfully extubated, and was significantly lower, 375 ml (7.0 ml/kg IBW), in the group for whom extubation failed. When the SBT started, and assist was removed, V_t _decreased very quickly and reached a plateau at significantly lower values in both groups. Compared with that of the successfully extubated group, V_t _remained lower at all times in the group for whom extubation failed.

During PSV_10_, f was 22 and 24 breaths/min in the groups of successful and failed extubation, respectively. Removal of PSV_10 _did not change f in the group of patients who were successfully extubated. In the extubation-failure group, f showed a tendency to increase during the early part of the SBT, such that f was significantly higher during T_1 _and T_5_, compared with the group with successful extubation. No significant differences in f were found between groups toward the end of the SBT.

During PSV_10_, V_E _was 9.9 L/min in the successfully extubated group, which was modestly but significantly higher than the V_E _of 8.4 L/min observed in the extubation-failure group. Except for a modest tendency for a decrease in VE during T_1_, V_E _remained stable throughout the SBT in both groups. The successfully extubated group maintained modest but significantly higher V_E _values throughout the majority of measurement points during the SBT.

The f/V_t _was 51 and 75 during the PSV_10 _period and was not different between groups. During the SBT, f/V_t _increased significantly in both groups and remained at values of about 60 to 70 (success) and 100 to 110 (failure) throughout the SBT.

Subanalysis of the patients for whom extubation failed revealed no significant differences for V_t_, f, V_E_, and f/V_t _between those whose SBT failed (*n *= 6) and those who were extubated but required reintubation, NIV, or died within 48 hours (*n *= 11).

### Blood gases

During PSV_10 _and throughout the SBT, bicarbonate in the group for whom extubation failed was elevated and significantly higher than that in the group that was successfully extubated (Table 2).

**Table 2:** Arterial blood gas and hemodynamic variables pressure support ventilation with 10 cm H_2_O above PEEP of 5 cm H_2_O (PSV_10_) and spontaneous breathing trial (SBT)

	Extubation outcome	PSV_10_		T_5_		T_end_	
		Mean	SD	Mean	SD	Mean	SD
pH	Success	7.45	0.05	7.46	0.05	7.47	0.08
	Failure	7.46	0.06	7.44	0.08	7.46	0.05
							
PaCO_2, _mm Hg	Success	36.4	7.6	37.3	8.5	35.9	8.7
	Failure	40.9	9.2	45.9^ab^	14.2	41.9^a^	9.3
							
HCO_3, _m*M*	Success	25.2	6.4	26.9^b^	5.3	26.0	5.5
	Failure	29.2^a^	4.8	30.8^a^	4.4	30.0^a^	4.3
							
PaO_2_/F_i_O_2_	Success	282.6	71.7	302.3	103.8	299.3	93.7
	Failure	257.9	81.3	249.0	79.3	243.5	91.1
							
HR, beats/min	Success	89.8	11.9	90.9	11.8	91.1	11.4
	Failure	95.9	12.1	95.4	11.3	98.0	13.0
							
MBP, mm Hg	Success	88.8	10.6	90.6	12.4	90.5	13.1
	Failure	83.9	7.4	82.2	9.8	89.4	16.1

Values are presented as mean ± standard deviation (SD). T_5 _and T_end _indicate values obtained at 5 minutes and the last values obtained during the SBT with CPAP of 5 cm H_2_O. ^a^Differences (*P *< 0.05) between failure and successfully extubated groups at the same time point. ^b^Differences (*P *< 0.05) between PSV_10 _and other time points in the respective groups.

During PSV_10_, PaCO_2 _values were normal and not significantly different between the groups. During the SBT, the PaCO_2 _increased in the extubation-failure group and reached higher values than those in the successfully extubated group (Table 2).

pH was stable and not different between groups at any time. The PaO_2_/F_i_O_2 _showed a consistent but nonsignificant tendency for higher values in the successfully extubated group.

### Extubation predictability

**Figure 4:**
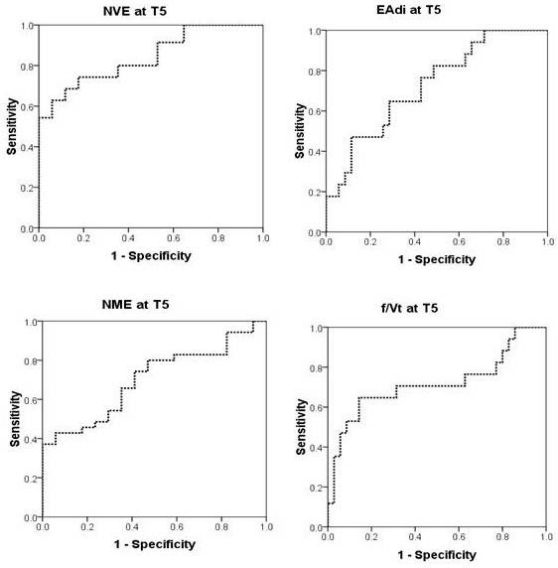
**Upper left panel shows the ROC curve (receiver operating characteristic) for neuroventilatory efficiency (NVE) to predict extubation success after 5 minutes of the spontaneous breathing trial (T_5_)**. Area under the curve (AUC) was 0.84 (*P *< 0.001). Upper right panel shows the ROC curve for diaphragm electrical activity (EA_di_) to predict extubation failure at T_5_. AUC was 0.73 (*P *< 0.009). Lower left panel shows ROC curve for neuromuscular efficiency (NME) to predict extubation success at T_5_. AUC = 0.70 (*P *< 0.02).The lower right panel shows the ROC curve for the ratio of breathing frequency and tidal volume (f/V_t_) to predict extubation failure at T_5_. AUC was 0.72 (*P *= 0.012).

NVE consistently demonstrated the highest values for predictability of extubation outcome throughout the SBT and could, according to the area under the ROC curve (AUC), be rated "good" for separating successful and failed extubation. Values for all time points and variables tested for extubation predictability are given in Table 3. Figure 4 shows the ROC curve for NVE predictability of extubation success at T5, having an AUC of 0.84 (*P *< 0.001) with a sensitivity of 0.69 and specificity of 0.88 at an NVE value of 24.1 ml/μV. Figure 4 also shows ROC curves obtained at T5 for EA_di _(predicting extubation failure, AUC = 0.73; *P *= 0.009), and NME (predicting extubation success, AUC = 0.70; *P *= 0.02). As a reference, we also show the ROC curve for the f/V_t _index (predicting extubation failure) having an AUC of 0.72 (*P *= 0.012). No statistically significant difference was found between the areas under the ROC curves for NVE, NME, EA_di_, and f/V_t_.

**Table 3:** Extubation predictability for the measured variables

				Cut-off point			
	Time	AUC	*P*	for analyzed	Sensitivity	Specificity	Youden index
				variables			
	T_1_	0.750	0.004	24.170	0.686	0.706	0.392
	T_5_	0.842	<0.001	24.070	0.686	0.882	0.568
NVE	T_10_	0.815	<0.001	20.120	0.743	0.765	0.508
(ml/µV)	T_15_	0.818	<0.001	33.620	0.600	1.000	0.600
	T_30_	0.861	<0.001	25.670	0.714	0.929	0.643
	T_end_	0.808	<0.001	22.210	0.771	0.824	0.595
	**Mean**	**0.816**		**24.977**	**0.700**	**0.851**	**0.551**

	T_1_	0.692	0.026	1.154	0.714	0.647	0.361
	T_5_	0.701	0.020	1.390	0.371	1.000	0.371
NME	T_10_	0.716	0.026	1.180	0.615	0.786	0.401
(cm H_2_O/µV)	T_15_	0.720	0.023	1.133	0.692	0.786	0.478
	T_30_	0.830	<0.001	0.879	0.914	0.667	0.581
	T_end_	0.745	0.005	1.139	0.800	0.647	0.447
	**Mean**	**0.734**		**1.146**	**0.684**	**0.756**	**0.440**

	T_1_	0.659	0.065	10.098	0.882	0.457	0.339
	T_5_	0.726	0.009	17.050	0.647	0.714	0.361
EA_di_	T_10_	0.718	0.012	19.740	1.000	0.543	0.449
(µV)	T_15_	0.718	0.013	9.655	1.000	0.486	0.486
	T_30_	0.780	0.002	14.400	0.786	0.743	0.529
	T_end_	0.743	0.005	14.410	0.706	0.743	0.449
	**Mean**	**0.724**		**14.226**	**0.837**	**0.614**	**0.436**

	T_1_	0.681	0.036	88.660	0.588	0.800	0.388
	T_5_	0.718	0.012	80.570	0.647	0.857	0.504
f/V_t_	T_10_	0.676	0.042	89.030	0.529	0.800	0.329
(breaths/min)/L	T_15_	0.714	0.015	72.790	0.688	0.771	0.459
	T_30_	0.716	0.019	64.540	0.786	0.629	0.415
	T_end_	0.745	0.005	69.620	0.765	0.686	0.451
	**Mean**	**0.708**		**77.535**	**0.667**	**0.757**	**0.424**

	T_1_	0.708	0.016	226.000	0.914	0.529	0.443
	T_5_	0.713	0.013	293.000	0.886	0.588	0.474
V_t_	T_10_	0.718	0.011	308.000	0.829	0.529	0.358
(L)	T_15_	0.745	0.005	302.000	0.943	0.626	0.569
	T_30_	0.740	0.009	302.000	0.829	0.571	0.400
	Tend	0.732	0.007	302.000	0.829	0.588	0.417
	**Mean**	**0.726**		**288.833**	**0.872**	**0.572**	**0.444**

	T_1_	0.645	0.106	6.714	0.893	0.471	0.364
	T_5_	0.654	0.074	6.960	0.829	0.529	0.358
VE	T_10_	0.677	0.040	7.165	0.829	0.529	0.358
(L/min)	T_15_	0.729	0.009	6.470	0.943	0.500	0.443
	T_30_	0.669	0.066	7.021	0.914	0.500	0.414
	T_end_	0.682	0.034	7.020	0.914	0.529	0.443
	**Mean**	**0.676**		**6.892**	**0.887**	**0.510**	**0.397**

	T_1_	0.666	0.055	26.240	0.588	0.829	0.417
	T_5_	0.649	0.084	26.820	0.529	0.829	0.358
f	T_10_	0.582	0.344	27.750	0.412	0.829	0.241
(breaths/min)	T_15_	0.596	0.273	29.330	0.375	0.886	0.261
	T_30_	0.600	0.278	27.570	0.500	0.771	0.271
	T_end_	0.618	0.169	27.560	0.529	0.800	0.329
	**Mean**	**0.619**		**27.545**	**0.489**	**0.824**	**0.313**

## Discussion

The major finding of this study is that a mixed population of critically ill patients for whom extubation fails are characterized by increased neural activation of the diaphragm as well as a reduced ability to generate inspiratory pressure and tidal volume for a given diaphragmatic neural activation. The present study also suggests that NVE has good predictive power to determine the outcome of extubation.

In the present study, the lower V_t_, higher f, and f/V_t_, as well as increased PaCO_2 _in the failure group, are all "classic" clinical signs associated with weaning failure, when compared with successful weaning [15,26]. Compared with the classic approach, the new measurement and indices (that is, EA_di_, NME, and NVE) were found to have more power to discriminate between groups for successful extubation and failed extubation for physiological reasons that are discussed later.

### EA_di_

EA_di _reflects the neural respiratory drive, and transesophageal measurements of EA_di _have been validated for reliability in healthy subjects and in patients with respiratory dysfunction [18-21,27-36].

In the present study, EA_di _was not different between the success and failure extubation groups at PSV_10_, suggesting that PSV provided sufficient assist to decrease neural respiratory drive in the failure group, despite a large difference in NVE. The higher bicarbonate level in the extubation failure group likely played a role in the lower V_E _in the failure group.

Our findings of a rapid increase in EA_di _reaching a plateau in both groups, with a higher plateau in the failure group, confirms previous work, demonstrating that inspiratory mechanical effort increases during SBT and increases more in patients for whom extubation fails [9-11]. This finding is in accord with previous studies demonstrating that 100 ms occlusion pressure (P0.1) was higher in patients for whom weaning failed [37,38].

The time until the EA_di _reached a plateau was shorter in the present study than previously reported for increases of mechanical effort when using T-piece [9,11]. This difference was likely due to differences in how the SBT was performed (T-piece or CPAP) [39,40].

Thus, our findings, along with previous findings, highlight that the neural respiratory drive is higher in patients for whom extubation fails.

### NME

A limitation of EA_di _is that it describes only neural respiratory drive, and it is not possible to ascertain whether an increase in EA_di _is due to diaphragm weakness, overcoming increased respiratory load, or to a response to increased metabolic demand.

Beck *et al*. [21] showed, in healthy subjects, that the EA_di _is linearly related to the diaphragm pressure generation up to 75% of maximum force. Finucane and Singh [41] showed that EA_di _is closely related to the diaphragm power output. Beck *et al*. [20] described that changes in transdiaphragmatic pressure closely resembled changes in EA_di _in patients with acute respiratory failure receiving mechanical ventilation. Given a relatively linear relation between EA_di _and diaphragm pressure generation, normalization of the inspiratory occlusion pressure to EA_di _provides a means to compare pressure-generating efficiency without the need to standardize effort.

In the present study, we measured the inspiratory P_aw _deflection during an inspiratory occlusion to reflect the inspiratory pressure generation. Previous studies have shown that this maneuver accurately reflects esophageal pressure deflections during both voluntary [42] and phrenic nerve-stimulated diaphragm contractions in intubated subjects [5,8].

It has been well established that the diaphragm contractile function decreases rapidly [4] to about 25% of normal values [3,5-8] in critically ill and mechanically ventilated patients. However, controversies still exist as to whether the inspiratory force is different between those for whom weaning fails and those who are successfully extubated [6,8]. In the present study, we found that the NME was significantly lower in the failure group, indicating a weaker diaphragm. This agrees with findings of Buscher [8], who compared twitch diaphragm force in patients successfully weaned shortly after surgery to patients for whom weaning failed and found lower twitch pressure in the failure patients. In contrast, Laghi *et al*. [6], who studied patients with similar duration of mechanical ventilation, did not find any difference in twitch diaphragm force between patients who were successfully extubated and for whom extubation failed.

Changes in FRC due to dynamic hyperinflation could have affected NME in the present study. Beck *et al*. [21] showed that an increase in volume from FRC to TLC reduces the transdiaphragmatic pressure by 60% for a given EA_di_. Sinderby *et al*. [43] showed that progressive dynamic hyperinflation in exercising COPD patients resulted in continuous increase of EA_di_, whereas the slope of the increase of transdiaphragmatic pressure decreased, and eventually plateau was reached, despite increased neural drive. In the present study, the higher number of patients with COPD in the extubation-failure group, prone to dynamic hyperinflation, may thus have been one contributing factor to the lower NME and NVE in the extubation-failure group.

Our findings of a lower NME in the extubation-failure group supports the concept that impaired pressure-generating capacity of the diaphragm, regardless of its origin, plays a role in the outcome of extubation.

### NVE

The NVE index (V_t_/EA_di_), reflects determinants of the volume generated (that is, the respiratory drive, diaphragm function, and respiratory load). In agreement with the notion that weaning failure is caused by respiratory demand exceeding the capacity of the respiratory muscles [12-16], NVE was the variable that demonstrated the largest difference between groups (50% lower NVE in the extubation-failure group) and best predictability for extubation outcome with the largest AUC (0.84). These findings agree with previous observations in COPD patients (22). Given the limitations of *post hoc *analysis, future prospective trials are required to show the predictive power of the NVE to determine extubation readiness.

The present study also showed that NVE is inversely correlated to age. This "negative" influence of age on the NVE was not surprising, given that respiratory mechanics, and lung and inspiratory muscle function decrease with age [44].

### Does NME or NVE discriminate fatigue during SBT?

Neither NME nor NVE changed throughout the SBT in either group. This result suggests that diaphragm contractility did not decrease and/or that load increased throughout the SBT. Previous studies suggested that the work of the diaphragm in nonassisted patients for whom extubation failed is such that diaphragm fatigue could develop [6,9,13]. This has been supported by early predictors of fatigue, such as muscle relaxation rate [14] and power spectrum shift of the EA_di _power spectrum [12]. Other studies, however, suggest that respiratory muscle contractile fatigue [6] and reduced muscle contractility [45] do not occur in weaning-failure patients. A possible explanation for these discrepancies is that the development of diaphragm fatigue would have a disastrous effect on the patient's survival and is therefore preceded by many changes in breathing strategies (for example, rapid shallow breathing and thoracoabdominal paradox) to prevent fatigue [6,11,14,21]. A dissociation between NVE and fatigue during strenuous breathing is also possible, as Luo *et al*. [28] showed, in healthy subjects, that diaphragm fatigue, expressed as >10% loss of diaphragm contractility, did not alter the NVE.

Hence, although not supportive of significant diaphragm fatigue development, our finding of constant NME and NVE over time throughout the SBT does not preclude the development of diaphragm contractile fatigue.

### Limitations of the method

The general population of critically ill patients in this study is skewed toward COPD and pneumonia (33 of 52) patients and does not represent specific disease states causing ventilatory failure. Future studies should focus on specific disease states.

The EA_di _in absolute units (µV) is affected by anatomic differences between subjects, where an increased distance between the electrodes in the esophagus and the crural diaphragm lowers the EA_di _amplitude [46]. Thus, unless this distance filtering is corrected for, some individual differences in EA_di_, NME, and NVE may pertain to anatomic deviations. However, given the size of the present study, occasional individual deviations should not affect the group-mean data.

Changes in recruitment of respiratory muscles other than the diaphragm may affect the NVE and NME. In healthy subjects who have a large respiratory muscle reserve and therefore the possibility to alternate between diaphragm and other inspiratory muscles, the errors of the NME and NVE indices to predict changes accurately in the diaphragm force-generating or ventilatory capacity can be expected to be of some magnitude unless the breathing pattern is controlled. In contrast, patients who have or are recovering from acute respiratory failure are characterized by very weak inspiratory muscles [47] and are not likely have enough reserve to alternate their inspiratory-pressure generation between different inspiratory muscle groups. Parthasarathy *et al*. [11] showed that the electrical activity of the rib cage and accessory inspiratory muscles increases early and more during a failing SBT. Although increased extradiaphragmatic muscle recruitment increases the amount of pressure and volume that will be delivered for a given EA_di_, it is unlikely that the diaphragm activity remains constant. It has been shown that the motor unit firing rate in healthy subjects increased more for the diaphragm than for nondiaphragmatic inspiratory muscles with increasing respiratory drive [48]. Sinderby *et al*. [27] provided evidence that changes in electrical activation of the diaphragm and intercostal muscles during near-maximal efforts are related in severe COPD patients. In the present study, nearly half of the changes in V_t _from T_1 _to T_end _were explained by changes of the EA_di _from T_1 _to T_end_, suggesting that the diaphragm is the major contributor to pressure and volume generation. In other words, it is not likely that diaphragm activation would remain constant when other extradiaphragmatic muscle activity increases.

It has been demonstrated that measures representative of breathing pattern and muscle efforts during weaning pertain to the method applied [39,40]. Numeric data from the present study should be interpreted with caution when compared with other approaches for SBT (for example, T-piece trial or PSV with or without PEEP). It should also be noted that differences in strategies to wean (CPAP, T-piece, PSV) may affect the results because they can have an impact on pressure/time product and hemodynamics differently [49,50].

Because of methodologic variability, methods to obtain and calculate EA_di _usually differ between investigators. Hence, comparison of numeric data from the present study may pertain only to other studies using the same standardized commercially available equipment. Compared with studies using the same equipment, the peak EA_di _observed during PSV in the present study agrees with previously reported data [33,51,52], suggesting that EA_di _values in patients with acute respiratory failure are in the same range. Values for the NME and NVE indices in the present study do not relate to infants. Future work on normalizing these indices to body weight is required to create a reliable index for all ages.

It can be argued that during conditions in which ventilatory drive is impaired or the diaphragm is weak, as are common in critical illness, EA_di _(also known as diaphragmatic EMG) may not reflect drive or strength. Theoretic descriptions of the relation between activation and EMG signal strength, as well as theoretic explanations for the relation between transdiaphragmatic pressure and global diaphragm activation, support that EA_di _reflects respiratory drive and its link to force generation [21]. Studies in animals have shown that the diaphragm EMG is related to phrenic nerve activity [53]. Studies in healthy subjects, patients with respiratory disorders, and mechanically ventilated critically ill patients show that EA_di _represents respiratory drive and that its amplitude and coupling to diaphragm force (transdiaphragmatic pressure) may vary because of, for example, anatomic variations and pathology [18-21,27-32,54-56]. Human motor-unit discharge rates are strongly associated with neural respiratory drive in both health and disease [57,58]. Rare conditions that rapidly affect neuromuscular transmission (for example, myasthenia gravis) can alter the ratio between central respiratory drive to the diaphragm and EA_di _[59]. Changes in muscle fiber action-potential velocity vary with diaphragm fatigue and temperature and alter the measured amplitude of the EA_di _relative to that of the respiratory drive [60]; however, these changes are not of a magnitude to explain the differences in EA_di _observed in the present study. Finally, data from studies using Neurally Adjusted Ventilatory Assist (NAVA) support that EA_di _has a close integration with chemoreceptors and vagal lung afferents during muscle unloading in acute respiratory failure [61]. Despite the evidence supporting that EA_di _is a reliable measure of respiratory drive, this research area is vast and requires further advancement.

In the present study, we applied a *post hoc *analysis to validate prediction of weaning success, but prospective trials are required to evaluate truly the accuracy of using EA_di_-based indices for prediction of weaning success.

## Conclusions

This study shows that a mixed group of critically ill patients for whom weaning fails are characterized by increased neural respiratory drive and impaired ability to convert neuromuscular activity into tidal ventilation, and this is at least in part due to inspiratory muscle weakness. Measurement of neuroventilatory efficiency (quantifying the imbalance between increased neural effort, respiratory load, and diaphragm weakness) can be valuable in clinical decision making about extubation readiness. Future studies should focus on specific disease states.

## Key messages

• Weaning failure is associated with increased neural respiratory drive and impaired ability to convert neuromuscular activity into tidal ventilation, and this is at least in part due to inspiratory muscle weakness.

• Measurement of neuroventilatory efficiency, quantifying the imbalance between increased neural effort, respiratory load, and diaphragm weakness, can be valuable in clinical decision making about extubation readiness.

## Abbreviations

AUC: area under the (ROC) curve; BP: blood pressure; COPD: chronic obstructive pulmonary disease; CPAP: continuous positive airway pressure; EA_di_: diaphragm electrical activity; f: respiratory frequency; f/V_t_: Weaning index based on ratio of breathing frequency and tidal volume; IBW: Ideal body weight; ICU: intensive care unit; NAVA: neurally adjusted ventilatory assist; NME: neuromechanical efficiency; NS: nonsignificant; NVE: neuroventilatory efficiency; P_aw_: airway pressure; PEEP: positive end-expiratory pressure; PSV: pressure support ventilation; PSV_10_: PSV of 10 cm H_2_O above a positive end-expiratory pressure (PEEP) of 5 cm H_2_O; ROC: receiver operating characteristic; SBT: spontaneous breathing trial; SIMV: synchronized intermittent mandatory ventilation; T_1_: minute 1; T_5_: minute 5; T_10_: minute 10; T_15_: minute 15; T_30_: minute 30; T_end_: the last data obtained during the SBT; V_E_: minute ventilation; V_t_: tidal volume.

## Competing interests

Arthur Slutsky is a consultant for Maquet Medical, the company that makes NAVA and is paid for this work. AS is Vice President, Research, at St. Michael's Hospital (SMH). SMH receives royalty payments from Maquet for NAVA. JB and CS have patented inventions related to neural control of mechanical ventilation. The license for these patents belongs to Maquet Critical Care. Commercial use of this technology provides financial benefit to JB and CS through royalties. JB and CS each owns 50% of Neurovent Research, Inc. (NVR), a research and development company that builds the equipment and catheters for research studies. NVR has a consulting agreement with Maquet Critical Care. JB and CS are inventors of NAVA and work for SMH. SMH receives royalty payments from Maquet for NAVA. The remaining authors have no competing interests to declare.

## Authors' contributions

LL was responsible for conception and design of the study, the acquisition, analysis, and interpretation of data, and drafting and revising the article for final approval of the version to be published. HL was responsible for the design of the study, acquisition and analysis of data, revising the article, and final approval of the version to be published. YY participated in the design of the study, acquisition and analysis of data, and revising the article for final approval of the version to be published. YH participated in the design of study, acquisition and analysis of data, and revising the article for final approval of the version to be published. SL participated in the design of study, acquisition and analysis of data, revising the article, and final approval for the version to be published. AS was involved with the conception and design of the study, interpretation of the data, revising the article for important intellectual content, and final approval of the version to be published. JB was involved with the conception and design of the study, analysis and interpretation of data, drafting and revising the article for important intellectual content, and final approval of the version to be published. CS was responsible for the conception and design of the study, analysis and interpretation of data, drafting and revising the article for important intellectual content, and final approval of the version to be published. HQ was involved with the conception and design of study, analysis and interpretation of the data, drafting and revising the article for important intellectual content, and final approval of the version to be published.
